# Comparison of a Novel Silicone Gel Wound Dressing vs Bacitracin After Follicular Unit Extraction Hair Transplantation

**DOI:** 10.1093/asjof/ojab051

**Published:** 2021-12-13

**Authors:** Isaac B James, David M Turer, Barry E DiBernardo

**Affiliations:** Department of Plastic Surgery, University of Pittsburgh Medical Center, Pittsburgh, PA, USA

## Abstract

**Background:**

Follicular unit extraction (FUE) hair transplantation subjects are excellent candidates to assess wound dressings. The wound surface area is large and adequately delineated to allow randomization, while in-patient split scalp designs allow patients to serve as their own controls.

**Objectives:**

This randomized, single-blinded, split-scalp comparison trial compares a novel, film-forming silicone gel—Stratamed (SM; Stratpharma AG, Basel, Switzerland)—to Bacitracin (Bac; McKesson Medical-Surgical Inc., Richmond, VA) in subjects undergoing FUE.

**Methods:**

Twenty subjects were randomized to receive SM and Bac on alternating sides of the scalp. Primary outcome measures included blinded clinician assessments of edema, erythema, crusting, healing response and outcome preference. Secondary measures included subject-reported assessments of pain and pruritis as well as FaceQ scores taken at post-FUE days two through six.

**Results:**

Twenty subjects were enrolled. Nineteen completed the trial. All subjects were non-smokers, and none had medical comorbidities expected to impact wound healing. An average of 1778 follicles per subject were harvested. No adverse events were reported, and all subjects healed by day 7. Healing response and outcome preference were significantly higher at day 1 in the SM group and by day 7, both groups were similar. There were no significant differences between groups for edema, erythema, or crusting. There were no significant differences between groups for subject-reported outcomes of pain, pruritis, or FACE-Q scores. When asked which product they preferred using, 44% of subjects preferred using SM versus 22% who preferred Bac.

**Conclusions:**

The SM wound dressing was well-tolerated in patients undergoing FUE. SM may speed the healing response in the early phase of wound healing.

**Level of Evidence: 2:**

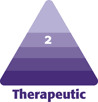

The number of new wound dressings with new technology claims coming onto the market is rapidly growing. For clinicians to assess these dressings, established measurement tools that can be used in an office-based or standard clinical setting are needed, without the resourcing of expensive researchers and equipment. Currently, the acute wound healing arena lacks these tools. In comparison, clinic-based chronic wound measurement tools are well established and published.^1-4^ To design tools that measure the quality and the speed of acute healing reliably is the most important component to first establish. This can then be implemented quickly into the routine day-to-day clinical setting for busy clinicians to assess new dressings and new dressing claims.

The acute wound healing process has 5 highly dynamic overlapping phases. Interventions that improve the early portions of the wound healing cascade (coagulation, inflammation, and epithelialization) benefit both surgeon and patient by reducing downtime, minimizing discomfort, and improving the patient experience. Furthermore, improvements in the early phases may also lead to more favorable outcomes in the latter half of the cascade (proliferation and remodeling) resulting in earlier final healing and improved scar quality. Stratamed (SM; Stratpharma AG, Basel, Switzerland) is a wound dressing which claims to reduce healing time, minimize wound signs and symptoms, reduce patient downtime, and improve scar quality. To measure these claims, we have devised an acute wound healing tool to compare against our standard treatment.

Patients undergoing follicular unit extraction (FUE) hair transplantation are ideal models to assess new measurement tools as well as new dressings. The wound surface area is sufficiently large, enabling treatment fields to be adequately delineated, randomized, and compared, whilst in-patient control allows for individual wound healing risk factors to be controlled. Downtime resulting from FUE is approximately 1 week and includes a donor site response of redness, swelling, crusting, itching, pain, and tightness.

Bacitracin (Bac; McKesson Medical-Surgical Inc., Richmond, VA), an antibiotic ointment, is used as the standard postoperative wound dressing in FUE. Since the worldwide launch of the antibiotic stewardship program over 10 years ago, hospitals and prescribers are constantly looking for strategies to minimize the usage and the adverse effects of antibiotics. SM would help promote this stewardship by eliminating the need for antibiotic ointments. SM is a full-contact, self-drying silicone gel dressing, which is bacteriostatic and indicated for open wounds and compromised skin. It can be used immediately over sutures, after FUE or overexposed dermis. Published evidence to date shows that SM speeds up the process of chronic wound healing.^5-7^ However, publications assessing its impact on acute wounds are limited.^8-11^

In the present study, we sought to compare SM with Bac, for healing response and downtime post FUE, using an objective, office-based acute wound healing metric. We hypothesized that, compared with Bac, using the SM dressing after FUE would reduce post-procedural downtime and donor site inflammation while also enhancing patient satisfaction with the procedure.

## METHODS

This study was conducted at New Jersey Plastic Surgery Centre, USA. It was in compliance with the Declaration of Helsinki, was approved by an ethics committee (Chesapeake IRB, Colombia, MD), and was registered on ClinicalTrials.gov under identifier number NCT03843996. Written informed consent was obtained for all patients before enrollment in the study, and all patients agreed to the use and analysis of their data. From January through December 2018, twenty apparently healthy participants aged 18 years or older, of any race and any Fitzpatrick skin type, with a desire to undergo FUE for hair restoration were screened and recruited. Treatment side assignment in the split scalp model was determined on an alternating basis as patients enrolled in the study. Participant exclusion criteria included patients who were immunocompromised, had inflammatory or autoimmune disorders, or were on medication that was known to affect the healing characteristics of the skin. Other exclusions included makeup, tattoos, or body piercings in the treatment field and patients who did not have adequate donor hair for transplantation. There were no significant changes to the protocol after trial commencement.

**Figure 1.**
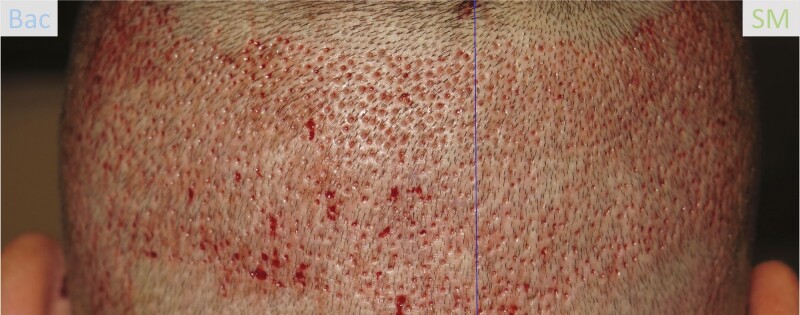
Whole scalp view illustrating split scalp design. A 41-year-old male patient who received Bacitracin treatment to his left scalp and Stratamed treatment to his right scalp following follicular unit extraction. He is shown here at his first follow-up visit (post-procedural day 2).

FUE transplantation was undertaken using the NeoGraft FU (Venus Concept, Weston, FL), using pneumatic controls to precisely extract complete hair follicles and then immediately transplanted to the desired areas of the scalp. During the procedure, 2000 to 2500 hairs were harvested and transplanted over 8 to 10 hours. The device left tiny punch holes in the donor area (Figure 1). Patients were randomly assigned to apply SM or Bac to either the left or right halves of the donor area on the back of the scalp twice per day for 7 days, as per the product information leaflets provided by the manufacturers. Patients were supplied with trial products free of charge.

Patients were evaluated in the office at baseline (immediately post-transplant) and then at post-procedural days 1 and 7 by a blinded clinical assessor. Because the order of enrollment did not correspond to the order in which procedures were performed, split scalp treatment assignment was in random effect. Four-point ordinal scale (ie, none, mild, moderate, or severe) was used for the assessment of donor site inflammation that included edema, erythema, and crusting. Overall, the healing response was also graded on a 4-point ordinal scale (ie, no healing, mildly healed, moderately healed, or completely healed). The blinded clinical assessor also recorded which side they thought had the better outcome. Wounds were also evaluated for infection and photographs were taken at each assessment. Self-reported measures were also collected daily for the first-week post-procedure. Patients were asked to rate their acute wound healing symptoms for edema, pruritus, pain/discomfort, and skin sensation for each side using a 4-point scale (ie, none, mild, moderate, or severe). Patients also completed the FACE-Q questionnaire (FACE-Q© 2013 Memorial Sloan-Kettering Cancer Center). General rating of patient product preference and compliance to the trial protocol was also recorded.

Data were analyzed using SPSS 24 statistical package (IBM, Armonk, NY). Descriptive analysis was done using standard statistical procedures. Student’s *t* test was used to assess normally distributed continuous variables. To determine statistical significance, *P* < 0.05 was used.

## RESULTS

### Demographics

Of the 20 patients enrolled, 19 completed the trial. One patient withdrew due to noncompliance with the protocol. Thirteen patients identified as Caucasian, 4 as Hispanic, 2 as South Asian, and 1 as black. Five patients had Fitzpatrick skin type 2, eight had type 3, four had type 4, one had type 5, and one had type 6. The average age was 39.6 years (standard deviation [SD] ±9.2; range, 22-57). Approximately, 94.7% (18/19) were male and 5.3% (1/19) were female. The average number of follicles harvested was 1778 (SD ±484). All patients were nonsmokers. No patients had medical comorbidities or were taking medications that would be expected to impair wound healing. Ten patients were assigned to receive SM on the left scalp and Bac on the right scalp, and 10 were assigned to receive SM on the right scalp and Bac on the left scalp. There were no donor site infections and no adverse events reported in either group. All patients were healed by their 7-day follow-up appointment. Demographic data are presented in Table 1.

**Table 1.** Demographics Table Describing the Sample Studied

Demographics	
Age (mean ± SD)	39.6 ± 9.2 years Range, 22-57
Sex	Male: 94.7% (18/19) Female: 5.3% (1/19)
Race	Caucasian: 63.2% (12/19) Black: 5.3% (1/19) South Asian: 10.5% (2/19) Hispanic: 21.1% (4/19)
Fitzpatrick skin type	I: 0% (0/19) II: 26.3% (5/19) III: 42.1% (8/19) IV: 21.1% (4/19) V: 5.3% (1/19) VI: 5.3% (1/19)
Smoking status	Nonsmoker: 100% (19/19)
Average follicles harvested (mean± SD)	1778 ± 484
Comorbidities	Diabetes:0% (0/19) Hypertension: 10.5% (2/19) Hyperlipidemia: 10.5% (2/19)

SD, standard deviation.

### Patient-Reported Outcomes

**Figure 2.**
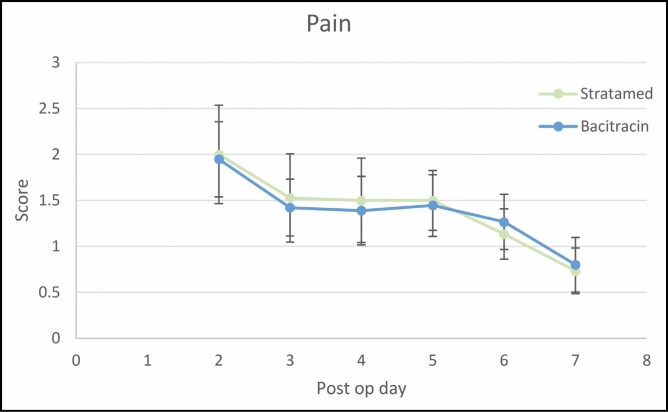
Patient-reported pain scores for each hemi-scalp at post-procedural days 2 through 7.

Patient-reported outcomes included daily self-assessments of pain, pruritus, and the FACE-Q on post-procedural days 2 to 7. Pain scores were not significantly different between groups at any time point but did significantly decrease from day 2 to day 7 in both SM and Bac cohorts (*P* = 0.009 and *P* = 0.01, respectively) (Figure 2).

**Figure 3.**
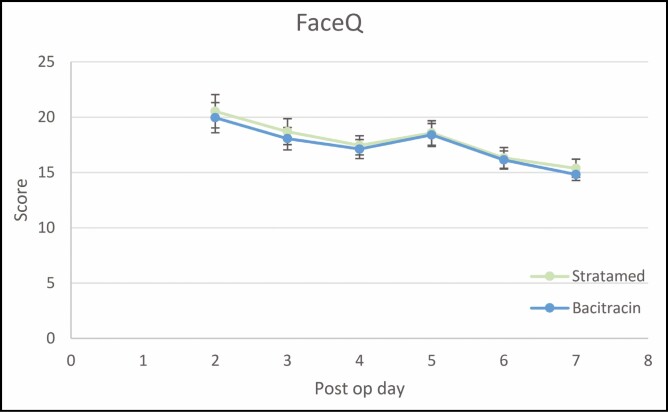
Patient-reported FACE-Q scores for each hemi-scalp at post-procedural days 2 through 7.

FACE-Q scores were also not significantly different between groups at any time point but did decrease from an average of 20.5 ± 6.6 at day 2 to 15.4 ± 3.3 at day 7 in the SM group (*P* < 0.001) and from 19.9 ± 5.9 at day 2 to 14.8 ± 2.2 at day 7 in the Bac group (*P* = 0.009) (Figure 3).

On post-procedure days 1 and 7, patient satisfaction with donor site treatment was assessed by asking them which side they thought had the better outcome at that time point. On post-procedure day 1, 65% of patients preferred the SM side, 25% of patients preferred the Bac side, and 10% of patients had no preference (Table 2). At post-procedure day 7, there was no difference in outcome preference as all donor sites had essentially fully healed (68% had no preference, 16% preferred SM, and 16% preferred Bac).

**Table 2.** Clinician Evaluation of Treatment Site Inflammation

	Day 1			Day 7		
	Bacitracin Mean (SD)	Stratamed Mean (SD)	*P*-value	Bacitracin Mean (SD)	Stratamed Mean (SD)	*P*-value
Edema	1.10 (0.64)	1.05 (0.60)	0.75	0.0 (0)	0.11 (0.32)	0.16
Erythema	1.10 (0.30)	1.00 (0.32)	0.16	0.32 (0.48)	0.26 (0.45)	0.33
Crusting	0.03 (0.15)	0.03 (0.10)	1.00	0.05 (0.12)	0.09 (0.15)	0.33
Healing response	0.40 (0.14)	0.54 (0.20)	**0.01**	1.0 (0)	0.98 (0.08)	0.33
Outcome preference	25%	65%	n/a	16%	16%	n/a

Standardized photographs of each hemi-scalp were shown to blinded clinician reviewers who evaluated the degree of edema, erythema, crusting, healing response, and overall outcome at each time point. SD, standard deviation.

**Figure 4.**
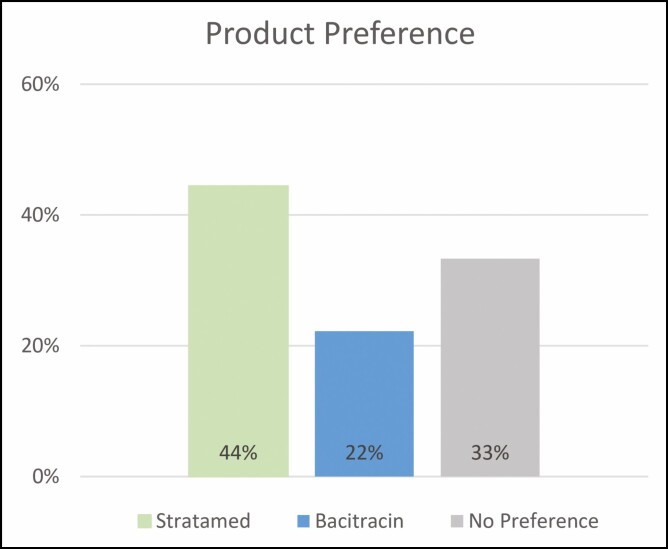
Patient-reported product preference assessed on post-procedural day 7.

On post-procedure day 7, patients were also asked which product they preferred using. Forty-four percent preferred using SM, 22% preferred using Bac, and 33% had no preference (Figure 4).

### Evaluation of FUE Donor Site Inflammation by Clinician Blinded to Intervention

Standardized photographs of each hemi-scalp were shown to blinded clinician reviewers who evaluated the degree of edema, erythema, crusting, healing response, and overall outcome at each time point. Edema was generally very mild but scores trended upward slightly in both groups on post-procedure day 1, fully resolving by day 7. There were no significant differences between SM and Bac groups at any time point (Table 2).

Similarly, erythema was minimal in both groups. Compared with immediate post-procedure, erythema scores increased on post-procedure day 1 in the Bac group (*P* = 0.010) and trended upward in the SM group (*P* = 0.096). However, there was no significant difference in erythema scores between SM and Bac groups (*P* = 0.16) on post-procedure day 1. Erythema had essentially resolved in all groups by day 7 (Table 2).

There was negligible crusting in either group at any time point. On post-procedure day 1, the average crusting score was 0.033 in both SM- and Bac-treated sites (*P* = 1.0). Similarly, on post-procedure day 7, the average crusting score was 0.087 on SM-treated sites and 0.052 on Bac-treated sites (*P* = 0.43) (Table 2).

**Figure 5.**
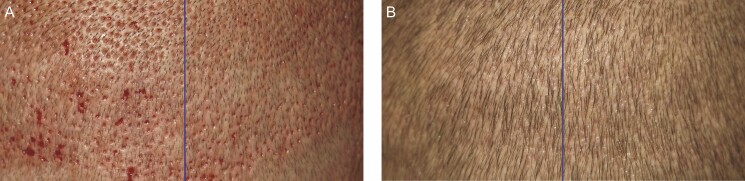
A 41-year-old male patient who received Bacitracin treatment to his left scalp and Stratamed treatment to his right scalp following follicular unit extraction. His course is representative of most other patients studied. Panel (A) demonstrates relative healing at his first follow-up visits (post-procedural day 2 in this case). Panel (B) demonstrates healing at his second follow-up visit (postprocedural day 6 in this case). Note that all wounds have essentially fully healed by day 6.

**Figure 6.**
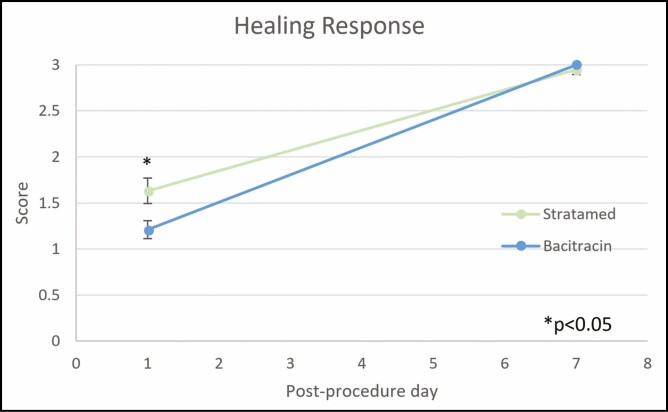
Clinician evaluation of healing response on days 1 and 7. Rated by a blinded clinician on a scale ranging from 0 for no healing to 3 for complete healing.

**Figure 7.**
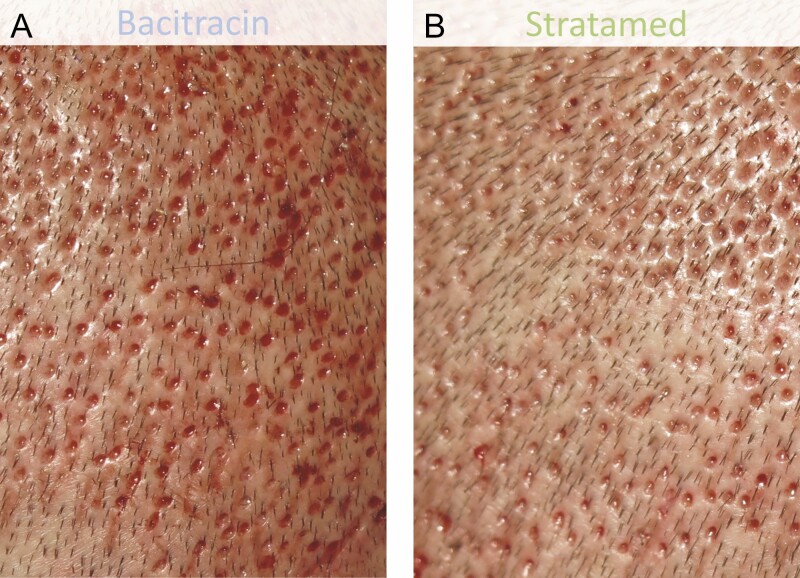
A 31-year-old male patient who underwent follicular unit extraction. He received Bacitracin treatment (A) to his left scalp and Stratamed treatment (B) to his right scalp. This photograph shows relative healing at his first follow-up visit (post-procedural day 1). Representative samples from respective treatment areas are shown.

**Figure 8.**
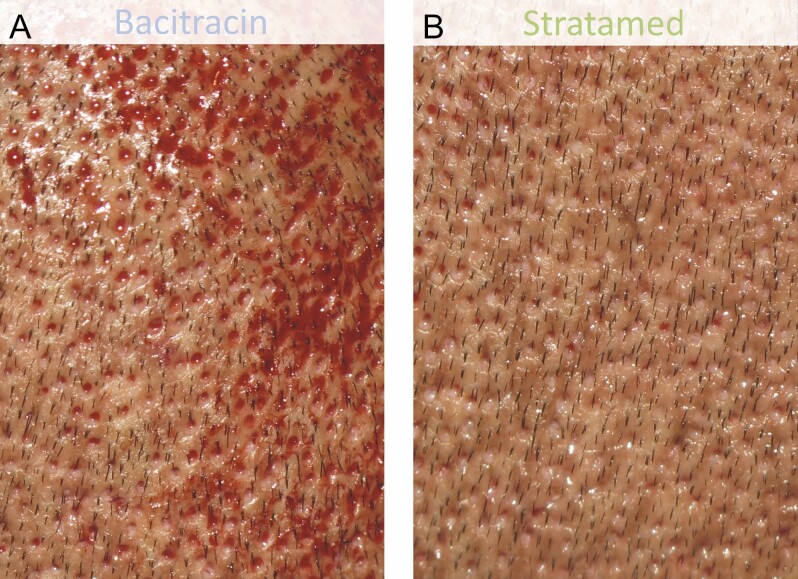
A 37-year-old male patient who underwent follicular unit extraction. He received Bacitracin treatment (A) to his right scalp and Stratamed treatment (B) to his left scalp. This photograph shows relative healing at his first follow-up visit (post-procedural day 1). Representative samples from respective treatment areas are shown.

**Figure 9.**
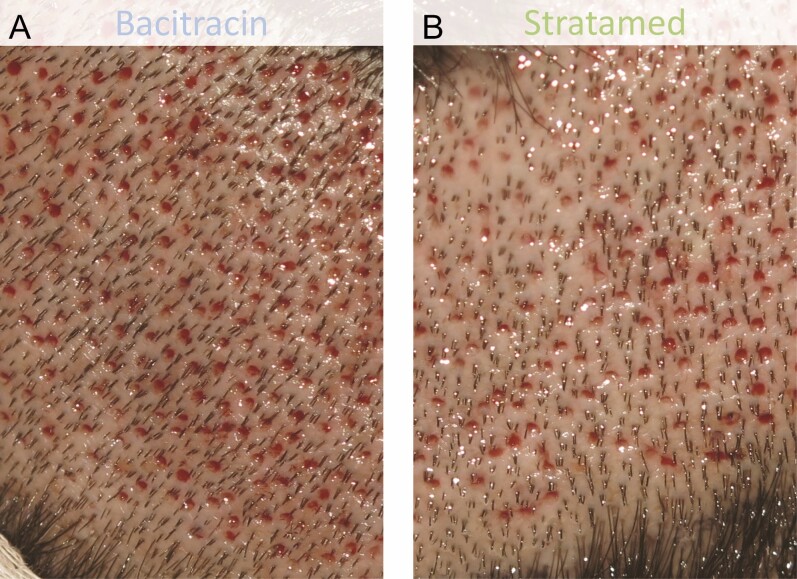
A 42-year-old male patient who underwent follicular unit extraction. He received Bacitracin treatment (A) to his right scalp and Stratamed treatment (B) to his left scalp. This photograph shows relative healing at his first follow-up visit (post-procedural day 2). Representative samples from respective treatment areas are shown.

**Figure 10.**
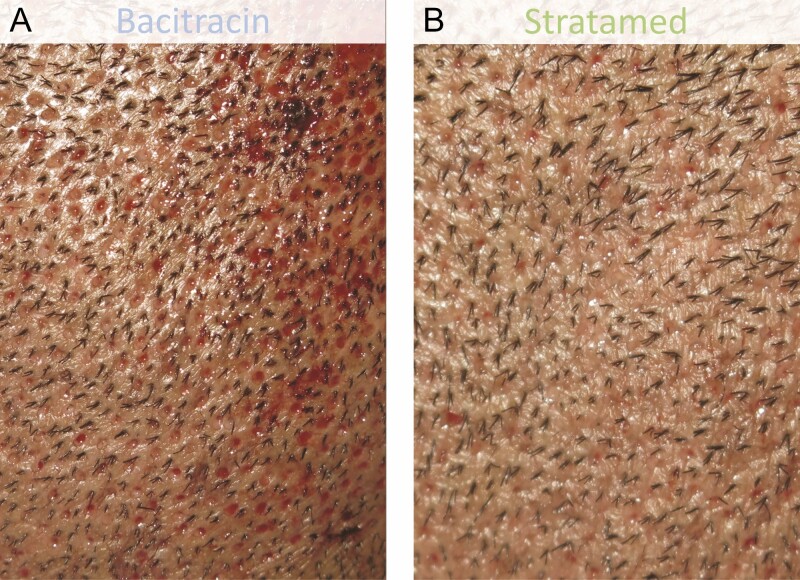
A 38-year-old male patient who underwent follicular unit extraction. He received Bacitracin treatment (A) to his left scalp and Stratamed treatment (B) to his right scalp. This photograph shows relative healing at his first follow-up visit (post-procedural day 2). Representative samples from respective treatment areas are shown.

**Figure 11.**
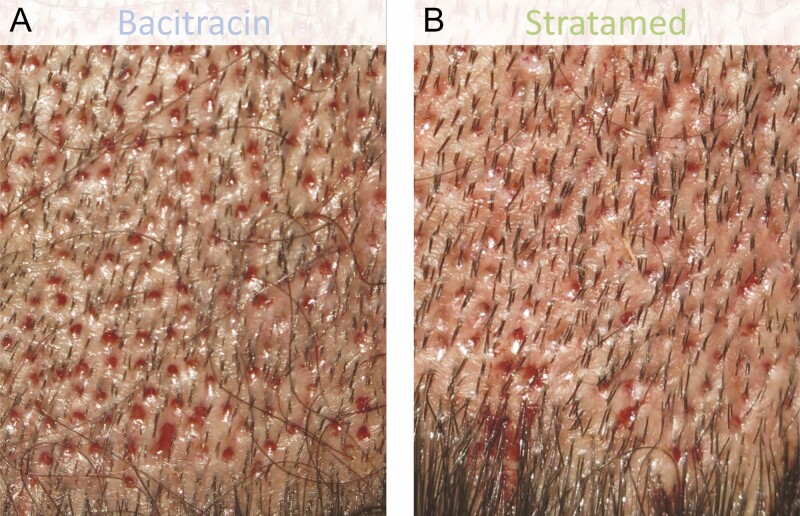
A 48-year-old female patient who underwent follicular unit extraction. She received Bacitracin treatment (A) to her left scalp and Stratamed treatment (B) to her right scalp. This photograph shows relative healing at her first follow-up visit (post-procedural day 2). Representative samples from respective treatment areas are shown.

**Figure 12.**
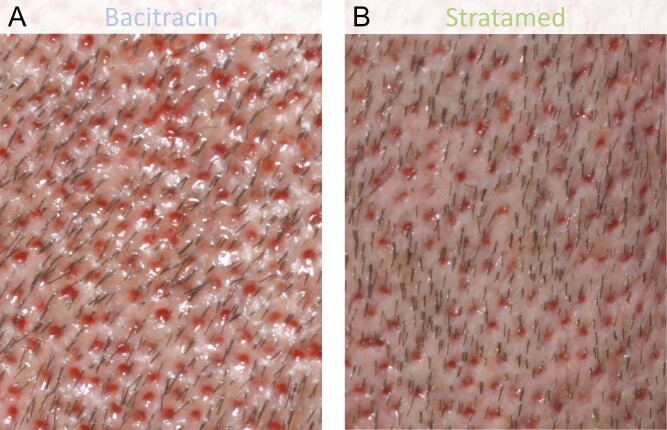
A 57-year-old male patient who underwent follicular unit extraction. He received Bacitracin treatment (A) to his left scalp and Stratame treatment (B) to his right scalp. This photograph shows relative healing at his first follow-up visit (post-procedural day 1). Representative samples from respective treatment areas are shown.

When looking at wound healing, however, SM showed significantly better healing on post-procedure day 1. By day 7, all wounds had essentially healed fully (Figure 5). On post-procedure day 1, the average healing response score was 0.54 in the SM group vs 0.40 in the Bac group (*P* = 0.014) (Figures 6-12). On post-procedure day 7, the healing response was 98.2% in the SM group and 100% in the Bac group (*P* = 0.33) (Figure 6).

**Figure 13.**
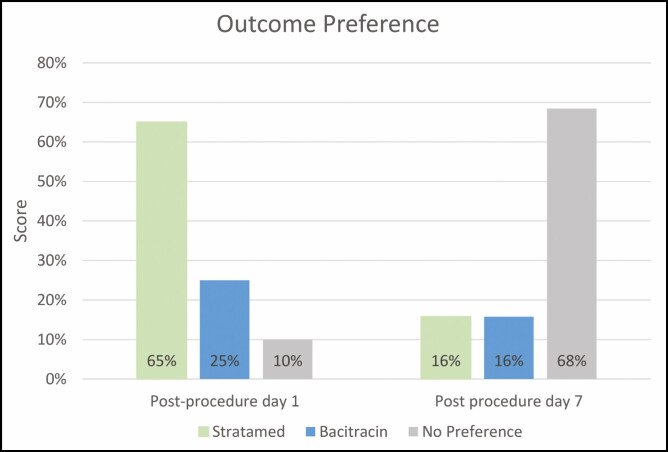
Patient-reported outcome preference on post-procedure days 1 and 7. Patients were asked which side had the better outcome at 2 time points: once during their post-procedure day 1 visit and once during their final post-procedure day 7 visit. On post-procedure day 1, most patients preferred the Stratamed side. On post-procedure day 7, most patients had no preference.

Similarly, when blinded clinicians were asked which outcome they thought was better, on day 1, the outcomes on the SM side were rated better 65% of the time compared with 25% on the Bac side. Approximately, 10% were rated as no difference. On day 7, 68% saw no significant difference between sides, while the SM side was preferred 16% of the time and the Bac side was preferred in the remaining 16% (Figure 13).

## Discussion

In this study, both SM and Bac groups recovered fully as expected. However, there appear to have been differences in the rate of healing. On day 1, SM had greater healing response and outcome preference as rated by independent blinded clinicians. Notable differences between groups can be seen in Figures 7-12. However, by day 7, both groups were essentially fully healed, and these differences were no longer evident (Figure 5). Meanwhile, there simply was not much erythema, edema, or crusting at any time point after FUE. As a result, there are no major differences between Bac and SM with respect to these metrics.

SM has several other advantages over Bac beyond hastening recovery. There is the well-known issue of contact dermatitis which occurs in many patients following Bac use. Rarely, Bac has even been associated with anaphylaxis and Stevens Jonson Syndrome.^12,13^ This has led to suggestions to avoid routine usage on clean surgical wounds.^14,15^ To date, SM has no such issues, and other silicone dressings have been well tolerated on the skin. Moreover, SM avoids concerns of promoting bacterial resistance or significantly altering the skin microbiome.

The natural history of wound healing following FUE is a rapid recovery with minimal intervention. However, the number and surface area of wounds provide a great model to gauge the effectiveness of wound care treatments as it allows a randomized split scalp model. The FUE model is best suited for assessing treatments for relatively minor wounds where scarring is not a major consideration. In this paradigm, the goals are to increase patient comfort and speed up wound healing. Recovery is expected relatively quickly with or without intervention, so the focus is really the acute phase of healing: from injury through epithelialization. This occurs very quickly in the FUE model.

To step back and look at longer-term wound healing metrics, such as scar maturation, would require a different model, such as intra-patient comparisons on abdominoplasty incisions^16^ or the Northwestern abdominoplasty scar model.^17^ These models better capture the subacute phase of wound healing and provide a greater wound healing challenge to prolong the healing curve. Likewise, the downside of these slower models is that it takes more time to perform. As such, it makes sense to first evaluate a fast model, such as FUE, to rapidly examine the acute phase of healing, and then move on to a model with a slower healing curve to evaluate subacute and long-term outcomes.

The limitations of this study must be recognized. Due to the model utilized, this study was targeted only to evaluate the impact of SM on the acute phase of wound healing in relatively minor wounds. This model does not evaluate longer-term outcomes, such as scarring. It also does not evaluate utility for large wounds or incisions. Moreover, patients comprising this study were primarily of Fitzpatrick skin types 2, 3, and 4. As such, it is possible that patients with other skin types not adequately represented in our sample would have different responses.

Additionally, due to the rapid nature of healing after FUE, we did not fully capture the entire healing curve with our study design. On day 1, when healing was still ongoing, the difference between SM and Bac was, at least subjectively, rather dramatic. However, by our next clinician evaluation on day 7, all wounds in both groups had essentially healed fully. In future studies utilizing an FUE split scalp model, we would recommend focusing on earlier postoperative follow-ups (ie, days 1, 2, 3, and 5) to better capture differences between groups. Although we only successfully captured a single time point before complete healing, knowledge of the time to healing after FUE is very important for refining the model for future use where it could be applied to a wide array of wound healing studies.

## Conclusions

The split scalp model provides a well-controlled way to compare wound dressings. For FUE donor sites, the SM wound dressing was well tolerated and had high patient satisfaction. Although both groups reached a similar 7-day endpoint, SM may provide a faster healing response in the early phase of wound healing which could decrease patient downtime after the procedure.
